# *Epilepsia open*—October 2024 announcements

**DOI:** 10.1002/epi4.13059

**Published:** 2024-10-04

## ILAE CONGRESSES

Translational Epilepsy Summer School 2024

21–25 October 2024

Subang Jaya, Malaysia

ASEPA SEEG Workshop and DIXI SEEG Course

October 30 to November 3, 2024

Bangkok, Thailand

Pediatric Epilepsy Surgery: From basics to advancements

November 7–10, 2024

Cochin, India

7th East Mediterranean Epilepsy Congress

12–15 December 2024

Baghdad, Iraq

## 2025

14th ILAE School on Pre‐Surgical Evaluation for Epilepsy and Epilepsy Surgery

January 20–24, 2025

Brno, Czech Republic

15th Asian & Oceanian Epilepsy Congress

February 20–23, 2025

New Delhi, India

5th African Epilepsy Congress

May 1–31, 2025

Africa

XVIII Workshop on Neurobiology of Epilepsy (WONOEP 2025)

August 25–29, 2025

Portugal

36th International Epilepsy Congress

August 30 to September 3, 2025

Lisbon, Portugal

## 2026

16th European Epilepsy Congress

5–9 September 2026

Athens, Greece

## ILAE WEBINARS

Approach to Difficult to Treat Epilepsies

3 October 2024

Webinaire éducatif sur l'épilepsie en français

8 October 2024

How to talk about SUDEP

23 October 2024

ILAE e‐Forum: Optimal timing for epilepsy surgery

November 25, 2024

## OTHER CONGRESSES

Chirurgie de l’epilépsie

1–2 October 2024

Tunisia

26èmes Journées Françaises de l'Epilepsie

October 8–11, 2024

Marseille, France

Epilepsy Training Course

October 10–11, 2024

Medan, Indonesia

Cours épilepsie et EEG pour les médecins de première ligne

October 11–12, 2024

Sfax, Tunisie

Cairo – Epilepsy Workshop

October 16, 2024

Cairo, Egypt

Epilepsy Training Course

October 18–20, 2024

Douala, Cameroon

2ème Journée de la Société Malienne des Neurosciences

19 October 2024

Bamako, Mali

2024 ILAE British Branch Annual Scientific Meeting

October 21–23, 2024

Liverpool, UK

SEEG Course 2024

October 31 to November 2, 2024

Thailand

2nd Annual Canadian Paediatric SEEG Conference

November 1–3, 2024

London, Ontario, Canada

Epilepsy Society of Australia Annual Scientific Meeting 2024

November 6–8, 2024

Hobart, Tasmania, Australia

2nd Advanced Course on the Pharmacological Treatment of Drug Resistant Epilepsies (DRE): From Rare to Common Epilepsies

November 8–10, 2024

Palma de Mallorca, Spain

2024 ILAE British Branch Clinical Epilepsy Course for Doctors in Training

November 9, 2024

Birmingham, UK

Paediatric Epilepsy Training

21 November 2024

Zambia

Encephalitis 2024

December 2–3, 2024

London, UK & Online

Second Symposium for Libyan Scientific Society for Epilepsy

6–7 December 2024

Tripoli, Libya

AES 2024 Annual Meeting

December 6–10, 2024

Los Angeles, California, USA

IBRO/ILAE Epilepsy School

December 9–12, 2024

Kinshasa, Democratic Republic of Congo

## 2025

1er Congreso Latinoamericano de Epilepsias Genéticas 2025

January 8–10, 2025

Santiago, Chile

19th World Congress on Controversies in Neurology

March 20–22, 2025

Prague, Czech Republic

AD/PD™ 2025 International Conference on Alzheimer's and Parkinson's Diseases and related neurological disorders

April 1–5, 2025

Vienna, Austria & Online

International Congress on Structural Epilepsy & Symptomatic Seizures 2025

April 2–4, 2025

Gothenburg, Sweden

International Conference for Technology and Analysis of Seizures (ICTALS 2025)

2–6 June 2025

Montreal, Canada

11th Congress of the European Academy of Neurology

June 21–25, 2025

Sevilla, Spain

21st San Servolo Advanced Epilepsy Course

21 July–1 August 2025

San Servolo (Venice), Italy

19th European Congress for Clinical Neurophysiology

September 9–12, 2025

London, UK

## 2026

18th Eilat Conference on New Antiepileptic Drugs and Devices

May 3–6, 2026

Madrid, Spain

